# Safety and tolerability of donepezil 23 mg with or without intermediate dose titration in patients with Alzheimer’s disease taking donepezil 10 mg: a multicenter, randomized, open-label, parallel-design, three-arm, prospective trial

**DOI:** 10.1186/s13195-019-0492-1

**Published:** 2019-05-01

**Authors:** Yun Jeong Hong, Hyun Jeong Han, Young Chul Youn, Kyung Won Park, Dong Won Yang, SangYun Kim, Hwa Jung Kim, Ji Eun Kim, Jae-Hong Lee, on behalf of the ODESA study (Optimal Dose Escalation Strategy to Successful Achievement of High Dose Donepezil 23 mg)

**Affiliations:** 1 Neurology, The Catholic University of Korea, Uijeongbu St. Mary’s Hospital, Uijeongbu, South Korea; 2 Neurology, University of Ulsan College of Medicine, Asan Medical Center, Seoul, South Korea; 3 Neurology, Dementia and Neurocognitive Center, Hanyang University College of Medicine, Myongji Hospital, Ilsan, South Korea; 4 Neurology, Chung-Ang University Hospital, Seoul, South Korea; 5 Neurology, Dong-A University College of Medicine, Busan, South Korea; 6 Neurology, The Catholic University of Korea, Seoul St. Mary’s Hospital, Seoul, South Korea; 7 Neurology, Seoul National University College of Medicine & Neurocognitive Behavior Center, Seoul National University Bundang Hospital, Seongnam, South Korea; 8 Preventive Medicine, University of Ulsan College of Medicine, Asan Medical Center, Seoul, South Korea; 9 Neurology, University of Ulsan College of Medicine, Gangneung Asan Hospital, Gangneung, South Korea

**Keywords:** Alzheimer’s disease, Safety, Tolerability, Dose-titration, High-dose donepezil

## Abstract

**Background:**

High-dose donepezil is currently prescribed for patients with Alzheimer’s disease (AD) who showed poor or waning response to a lower dose at the risk of increasing cholinergic side effects. However, the adverse events (AEs) depending on the method of dose escalation have not been clarified yet. This study aimed to find out whether dose titration before escalating to donepezil 23 mg is preferred. We investigated safety and tolerability of donepezil 23 mg during the first 12 weeks of dose escalation in patients with moderate to severe AD.

**Methods:**

This study was a 12-week, multicenter, randomized, open-label prospective trial. We included patients with moderate to severe AD who were treated with a stable dose of donepezil 10 mg/day. Patients were randomized into 3 groups according to the dose escalation method: 15 mg of donepezil for 4 weeks before escalating to 23 mg (group 1), 10 mg and 23 mg on alternate days for 4 weeks prior to escalation (group 2), and direct escalation to 23 mg (group 3). Safety analyses included incidence, severity, timing of AEs, relationship to the study drug, and premature study discontinuation due to AEs between the groups.

**Results:**

Among 175 enrolled, 110 patients completed the study. Baseline characteristics were similar among the groups. Using safety population (*N* = 160), cholinergic gastrointestinal symptoms including anorexia and nausea were the most common AEs and titration groups showed significantly fewer cases of nausea as compared with those in no-titration group.

**Conclusions:**

In this study, dose titration before escalating to donepezil 23 mg/day showed better safety in terms of cholinergic AEs. We suggest that dose titration during the first 4 weeks can be recommended for patients with moderate to severe AD.

**Trial registration:**

Clinicaltrials.gov, NCT02550665. Retrospectively registered on 15 Sep 2015.

**Electronic supplementary material:**

The online version of this article (10.1186/s13195-019-0492-1) contains supplementary material, which is available to authorized users.

## Background

In Alzheimer’s disease (AD), cholinergic neuronal loss occurs in the early stage and aggravates according to the disease progression. Hence, cholinergic deficit becomes more prominent in the advanced stage. Donepezil is a cholinesterase inhibitor and has a dose-related cognitive benefit in AD; however, a standard dose of 10 mg/day is known to inhibit only 20–40% of total cortical cholinesterase activities [1–3]. Earlier trials randomized to donepezil 5 mg/day, donepezil 10 mg/day, and placebo revealed cognitive benefits in patients with AD receiving higher dose of donepezil (10 mg/day) regardless of the stages, and even higher dose could bring further benefit considering there is still room for suppressing cholinesterase activity to the maximum. [3]. Based on this rationale, a higher dose of acetylcholinesterase inhibitor (AChEI) was called for in the advanced stage. Subsequently, donepezil 23 mg was approved for the symptomatic pharmacological treatment of moderate to severe AD following the study that showed cognitive benefits of donepezil 23 mg/day compared with 10 mg/day in a large, 6-month, phase 3 clinical trial [4]. In this trial, AD patients with moderate to severe AD who were on a stable dose of donepezil 10 mg/day were included and divided into two groups, a group of continuing donepezil 10 mg/day or a group of escalating donepezil to 23 mg/day; donepezil 23 mg/day demonstrated greater cognitive benefits on severe impairment battery score. Moreover, the cognitive benefits were more pronounced in patients with lower Mini-Mental State Examination (MMSE) scores, suggesting that AD patients with more advanced stages may require higher doses of an AChEI to perform an optimal response [5, 6]. However, the adverse events (AEs) were also increased, which would negatively influence drug compliance and limit the usefulness of high-dose donepezil [7–10]. Usually, dose titration is recommended to enhance drug adherence and reduce AEs in other situations; however, there is no consensus or scientific evidence about the dose titration in this circumstance. Considering that most AEs were reported during the first 4 weeks after escalating to high-dose donepezil [8], dose titration might be appropriate during the risky period. In addition, Asian population is reported to be more susceptible to cholinergic side effects, such as anorexia, nausea, vomiting, and dizziness [10, 11].

The primary objective was to investigate safety and tolerability of high-dose donepezil 23 mg during the first 12 weeks using dose titration in moderate to severe AD patients. The secondary objectives were to compare the incidence and severity of AEs between dose-titration and no-titration groups and to confirm whether the dose titration during the first 4 weeks would be beneficial in terms of safety and tolerability.

## Methods

### Study design and procedures

This study was a randomized, multicenter, open-label, parallel-group, prospective clinical trial conducted in six centers in South Korea between December 2014 and August 2016. The study duration was 12 weeks, and the dose-titration duration was the first 4 weeks from the baseline. Patients with moderate to severe AD dementia were consecutively enrolled and randomized into three groups; groups 1 and 2 are dose-titration groups during the first 4 weeks using two different titration methods, and group 3 is no-titration group with direct escalation to 23-mg donepezil. Concomitant use of cognitive enhancers (i.e., choline alfoscerate, *Ginkgo biloba*, or acetyl-l-carnitine) or memantine was permitted but maintained at a stable dose during the study period. Other AChEIs and investigational products for cognition were prohibited during the study period or should have been discontinued for ≥ 12 weeks before screening. Medications that might cause gastrointestinal (GI) problems or cholinergic side effects were permitted (including antiplatelet agents, antihypertensive drugs, antidepressant drugs including selective serotonin receptor inhibitors, and cholinergic agonists), but should be taken with a stable dose for at least 3 months prior to the screening and maintained during the study. Study drug was provided free of charge and was administered at any time of day. To determine drug compliance, unused drugs were counted and recorded at every visit in each center. The number of remaining drugs was divided by the number of initially prescribed drugs at every visit, and the mean drug compliance was measured.

### Patients

Patients with moderate to severe AD dementia were recruited. Our inclusion criteria were as follows: (1) age ranged between 45 and 90, (2) patients diagnosed with probable AD dementia according to the NIA-AA criteria [12], (3) patients who had been receiving donepezil 10 mg/day for at least 3 months prior to screening, (4) moderate to severe AD (MMSE [13], 0–20; clinical dementia rating (CDR) score [14], 2 or higher), (5) existence of a reliable caregiver who was sufficiently familiar with the patient to provide the investigator with accurate information, and (6) patient and caregiver agreed with the study participation. Patients were excluded if they had the following: (1) any neurological or psychiatric disorders that might cause dementias not related to AD (i.e., Parkinson’s disease, active epilepsy, normal pressure hydrocephalus, stroke, Huntington’s disease, schizophrenia, bipolar disorders); previous history of major depressive disorder was permitted; (2) history of participation in a clinical trial within 3 months prior to screening; (3) any severe or unstable medical disease that may prevent the patient from participating all study requirements (i.e., severe pulmonary, cardiovascular, GI, hematologic, endocrine, hepatic, or renal disease); (4) patients treated with other anti-dementia medications within 3 months (memantine combination with a stable dose of donepezil was allowed); (5) substance or alcohol abuse history within the past 5 years; and (6) previous history of intolerance or hypersensitivity to AChEIs. Written informed consent was obtained from the subject before the initiation of the study.

We divided patients into three groups according to the dose escalation method: 15 mg of donepezil for 4 weeks before escalating to 23 mg (group 1), 10 mg and 23 mg on alternate days for 4 weeks before escalating (group 2), and direct escalation to 23 mg (group 3).

The study protocol and informed consent form were reviewed and approved by the institutional review board of each center. The study was conducted in accordance with the Declaration of Helsinki and principles of Good Clinical Practice.

### Safety and tolerability outcome variables

Primary safety outcome variable was the incidence of treatment emergent adverse events of special interests (AESI). AESI included nausea, vomiting, diarrhea, anorexia, abdominal pain, headache, bradycardia, and weight loss. Nausea, vomiting, diarrhea, anorexia, abdominal pain, and headache were assessed by the patient’s and caregivers’ self-reports. Bradycardia was defined as a low pulse rate below 50 per minute at any time during the study period. Weight loss indicates ≥ 7% weight decrease compared with the baseline weight at any time during the study. Secondary safety outcome variables included dropout rates, mean drug compliance (number of actually taken drugs/total number of prescribed drugs), vital signs, changes in weight or electrocardiogram at week 12, blood laboratory findings at week 12 (including hematology, biochemistry, and electrolytes in each center), and any AE occurrence (incidence, symptoms, severity, and relationship with the study drug). The AE occurrence was recorded using both spontaneous reports from patients/caregivers at any time throughout the study period and open-ended questioning by study staff at all study visits. Severities of AEs were rated as mild, moderate, or severe: AEs causing minimal inconvenience and easily tolerated without interfering normal daily activities were rated as mild, AEs interfering with the participant’s daily activities/functioning were rated as moderate, and AEs interrupting the participant’s normal daily activities were rated as severe. Relationships between the study drug and AEs were rated as unrelated, possibly related, or probably related: AEs clearly not related to the study drug were considered to be unrelated, AEs which follow a reasonable temporal sequence from initiation of the study drug but could be caused by other factors were considered to be possibly related, and AEs which follow a reasonable temporal sequence from initiation of the study drug and confirmed by improvement on stopping the study drug and that could not reasonably explained by other known characteristics of the participant’s clinical status were considered to be probably related. We assessed physical examinations, weight, vital signs, concomitant medications, and AE at each study visit (baseline, weeks 4, 8, and 12). Laboratory tests were performed at baseline and week 12. Subjects who withdrew prematurely were asked to complete all end-point assessments at the time of early termination. A visit window for five calendar days was allowed for the follow-up visits.

### Sample size and randomization

Sample size was calculated under the assumption that incidence of AESI in group 1 would be 15% (95% confidence interval, 3–18%). Because there was no previous study that had investigated safety of donepezil 23 mg after dose titration, we exploratively presumed that the incidence would be lower than that of previous studies without dose titration [8, 9, 15]. As a result, 60 patients in each group were needed for our study without considering dropout rates as the study aimed to investigate safety and tolerability.

Patients were consecutively randomized in a 1:1:1 ratio using web-based stratified block randomization in each center. Stratification was based on gender and weight, whether a patient’s weight at baseline is below 55 kg or 55 kg or more, considering even distribution of gender and weight.

### Statistical analyses

Safety set population was defined as all randomized patients who had a baseline evaluation, took at least one study drug, and at least one post-baseline assessment, regardless of study completion or discontinuation. Per-protocol (PP) population was defined as patients who completed the study without any major protocol violations.

Baseline demographic and clinical characteristics were compared using the analysis of variance (ANOVA) or Kruskal-Wallis tests for continuous variables according to the normal distribution patterns. Dunnett’s methods were used for post hoc analyses. Chi-square tests were used for categorical variables in both safety set population and PP population. Safety outcomes were analyzed in the safety set population. Group comparisons for incidences of AEs were performed using chi-square tests or Fisher’s exact tests. Odds ratio of each AE was assessed using binary logistic regression analysis. Significance for all tests was set at *α* = 0.05, two-tailed. All statistical tests were conducted using SAS version 9.4 (SAS Institute Inc., Cary, North Carolina, USA).

## Results

### Baseline demographics and clinical findings

**Fig. 1:**
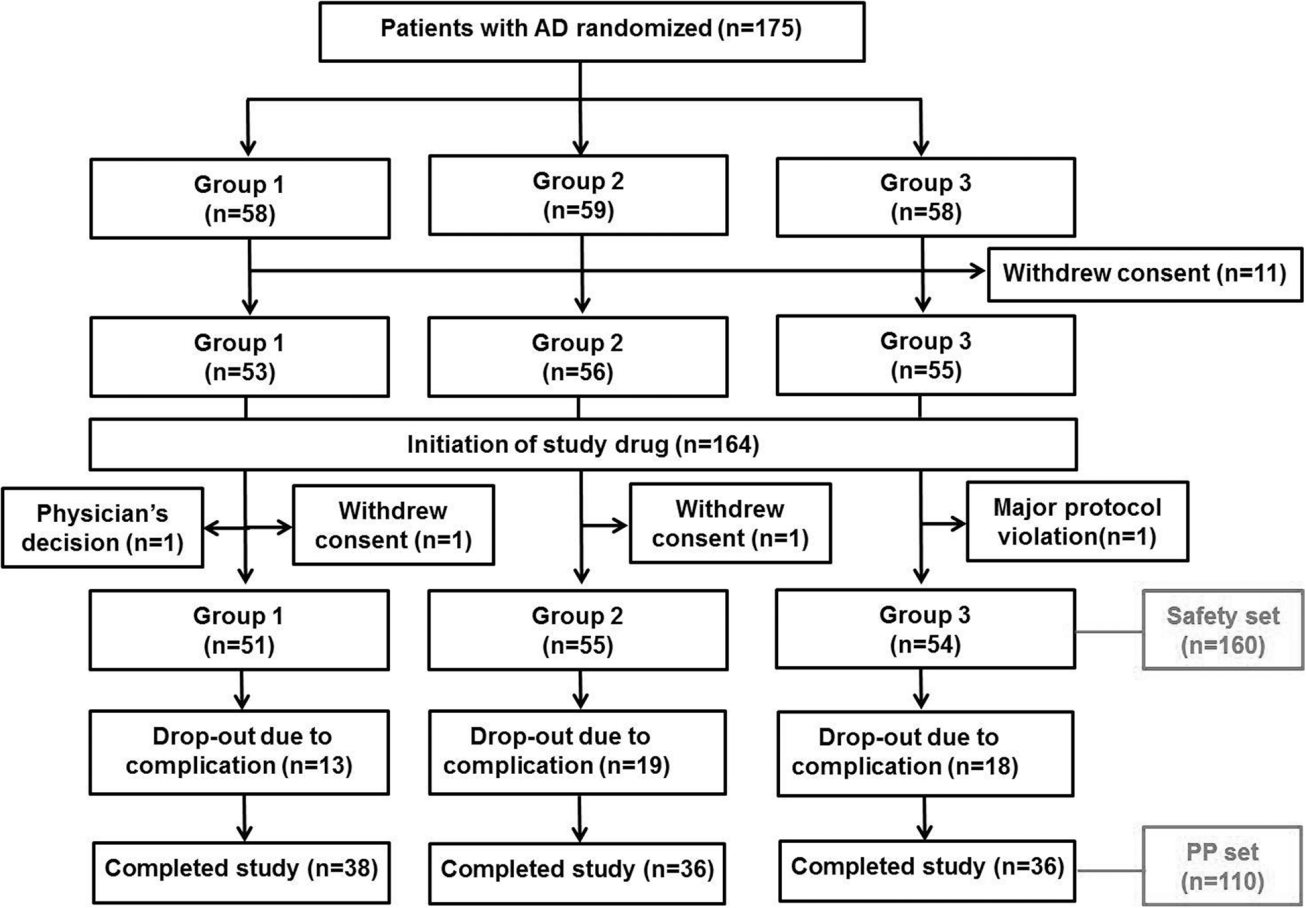
Study flowchart

Among 175 patients who were eligible and randomized, 11 patients withdrew consents after randomization, leaving 164 patients who initiated the study drug. During the study period, 4 patients discontinued the study drug without any follow-up evaluations due to physician’s decision (*n* = 1), major protocol violation (*n* = 1), and consent withdrawals (*n* = 2). The other 50 patients dropped out from the study due to AEs (see Fig. 1). Finally, 110 patients completed the study without any major protocol violation, being included in the PP population (see Additional file 1: Table S1). Study completers were not different from dropout patients in terms of baseline characteristics (Additional file 1: Table S2). The 160 AD patients who underwent randomization, took at least one study drug, and underwent at least 1 follow-up evaluation are included in the safety set population. We mainly show analyses using the safety set population because this study focused on safety and tolerability of the study drug. Baseline demographics and clinical status were similar among the 3 groups (*p* > 0.05, Table 1). Post hoc analyses did not show any differences between the groups in regards of baseline characteristics. Mean age was 75.2 years old, mean MMSE score 13.7, and mean general deterioration scale score 5 in the safety set population.

**Table 1:** Baseline demographics and clinical characteristics of the subjects (safety population)

Variables	Group 1 (*n* = 51)	Group 2 (*n* = 55)	Group 3 (*n* = 54)	*p*
Age, years	74.8 ± 8.2	75.0 ± 9.6	75.8 ± 8.2	0.867
Female, %	60.8%	60.0%	63.0%	0.958
Education, %low/med/high	49/25.5/25.5	56.4/21.8/21.8	51.9/29.6/18.5	0.834
K-MMSE	12.9 ± 5.3	13.7 ± 4.3	14.4 ± 4.7	0.247
CDR	1.6 ± 0.6	1.7 ± 0.7	1.7 ± 0.5	0.877
GDS	4.9 ± 0.6	4.8 ± 0.7	4.9 ± 0.7	0.679
Body weight, kg	57.8 ± 8.7	59.2 ± 11.4	57.5 ± 9.9	0.628
BMI	23.9 ± 2.7	24.1 ± 3.6	23.6 ± 3.1	0.752
Duration of donepezil, years	1.8 ± 2.1	1.9 ± 2.0	2.0 ± 2.1	0.791
History of side effect d/t donepezil, %	3.9%	7.3%	3.7%	0.733
Hypertension, %	43.1%	58.2%	40.7%	0.147
DM, %	19.6%	27.3%	25.9%	0.661
Hyperlipidemia, %	33.3%	38.2%	35.2%	0.895
Brain injury, %	7.8%	7.3%	9.3%	0.937
Concomitant memantine	16/51, 31.4%	14/55, 25.5%	14/54, 25.9%	0.753

Educational level up to 6 years (elementary school) was rated as low, up to 12 years (graduation high school) was rated as medium, and above 12 years was rated as high

*K-MMSE* Korean version of Mini-Mental State Examination, *CDR* clinical dementia rating, *GDS* general deterioration scale, *BMI* body mass index, *DM* diabetes mellitus

### Safety and tolerability

Treatment emergent adverse events (TEAEs) using the safety set population are shown in Table 2. Overall, 101/160 (63.1%) of the safety population experienced ≥ 1 TEAE during the study period. Incidences of AESIs were 41.9% in all safety population (group 1, 20/51, 39.2%; group 2, 22/51, 40.0%; group 3, 25/54, 46.3%) and did not differ among the three groups. Among the AESIs, bradycardia was not reported in our study (Table 2). Anorexia, dizziness, nausea, vomiting, generalized weakness, and diarrhea were the most frequently reported TEAEs (over 10% of total patients) across all groups. Most cases of TEAEs were mild in severity (mild AEs 149/160 (93.12%); Table 2). The most common TEAE in the groups 1 and 2 was anorexia, while nausea was the most common TEAE in the group 3 (no-titration group). Nausea and/or vomiting, dizziness, anorexia, and generalized weakness were the most common TEAEs that had caused premature study discontinuations (Table 2). There were no clinically important abnormalities/changes in laboratory values, vital signs, or electrocardiogram findings between baseline and end of the study (data not shown).

**Table 2:** Treatment emergent adverse event profiles during study period (safety population)

Variables, incidence (95% CI)	Group 1, 15 mg (*n* = 51)	Group 2, 10/23 mg (*n* = 55)	Group 3, no titration (*n* = 54)	*p* value G1,2 vs G3
AE_Total	28/51, 54.9% (40.34~68.87)	37/55, 67.3% (55.32~79.34)	36/54, 66.7% (52.56~78.94)	0.34
AESI	20/51, 39.2% (25.83~53.87)	22/51, 40.0% (27.02~54.09)	25/54, 46.3% (32.63~60.39)	0.72
AE-GI symptoms	17/51, 33.3% (20.73~47.89)	20/55, 36.4% (23.85~50.47)	24/54, 44.4% (30.88~58.56)	0.48
Anorexia	10/51, 19.6% (9.82~33.11)	14/55, 25.5% (14.71~39.05)	12/54, 22.2% (12.03~35.58)	0.77
Dizziness	4/51, 7.8% (2.16~18.82)	8/55, 14.5% (6.50~26.66)	12/54, 22.2% (12.03~35.58)	*0.07*
Nausea	5/51, 9.8% (3.26~21.41)	5/55, 9.1% (3.02~19.97)	13/54, 24.1% (13.51~37.67)	0.04*
Vomiting	4/51, 7.8% (2.16~18.82)	7/55, 12.7% (5.26~24.45)	9/54, 16.7% (7.94~29.33)	0.39
Generalized weakness	4/51, 7.8% (2.16~18.82)	8/55, 14.5% (6.50~26.66)	6/54, 11.1% (4.18~22.62)	0.97
Weight loss	4/51, 7.8% (2.16~18.82)	5/55, 9.1% (3.02~19.97)	3/54, 5.6% (1.18~15.45)	0.81
Diarrhea	2/51, 3.9% (0.47~13.43)	5/55, 9.1% (3.02~19.97)	6/54, 11.1% (4.18~22.62)	0.41
Dyspepsia	0/51, 0% (0~6.98)	5/55, 9.1% (3.02~19.97)	3/54, 5.6% (1.18~15.45)	1.00
Urinary frequency	3/51, 5.9% (1.23~16.24)	5/55, 9.1% (3.02~19.97)	0/54, 0% (0~6.60)	*0.05*
Increased neuropsychiatric sx.	0/51, 0% (0~6.98)	2/55, 3.6% (0~6.60)	3/54, 5.6% (1.18~15.45)	0.34
Insomnia	1/51, 2.0% (0.1~10.45)	3/55, 5.5% (1.14~15.12)	0/54, 0% (0~6.60)	0.30
Headache	0/51, 0% (0~6.98)	0/55, 0% (0~6.49)	3/54, 5.6% (1.18~15.45)	*0.07*
Tremor	1/51, 2.0% (0.1~10.45)	1/55, 1.8% (0~9.72)	1/54, 1.9% (0~9.72)	1.00
Dropout d/t AEs	13, 25.5%	19, 34.5%	18, 33.3%	0.69
AESI, severity (mild/moderate/severe)	19/1/0	18/4/0	19/5/1	0.19
Most common AEs leading to discontinuation	Anorexia 7.8%, nausea/vomiting 5.9%, dizziness 3.9%	Anorexia 10.9%, nausea/vomiting 7.3%, generalized weakness 7.3%	Nausea/vomiting 18.5%, dizziness 14.8%, anorexia 7.4%	n/a

*Significantly different incidences between titration and no-titration groups. AE-GI symptoms include anorexia, nausea, vomiting, and diarrhea. AESI includes nausea, vomiting, diarrhea, anorexia, abdominal pain, headache, bradycardia, and weight loss. Severities of AEs were rated as mild, moderate, or severe

*AE* adverse event, *AESI* adverse events of special interests

In most TEAEs, the incidences were numerically the lowest in the group 1 and the highest in the group 3, although not statistically significant. When comparing dose-titration groups (groups 1 and 2) with no-titration group (group 3), titration groups showed significantly fewer cases of nausea (see Table 2). Dizziness and headache also showed trends of lower occurrences in the titration groups (0.05 < *p* < 0.1). Using both PP set (*n* = 110) and safety set (*n* = 160), dropout rates due to AEs, severities of AEs, and the incidence of AESI were not statistically different among the groups (Table 2). However, dropout rates were numerically the lowest in the group 1 (25.5%) and the highest in the group 2 (34.5%). Severities of AEs were mostly mild regardless of the groups; moderate to severe AEs were reported in 1 patient of group 1, 4 patients of group 2, and 6 patients of group 3 (Table 2).

In Table 3, adverse drug reactions (ADRs) in each group are listed. ADRs indicate TEAEs that were reported to be possibly or probably related with the study drug. Anorexia, dizziness, nausea, vomiting, and generalized weakness were the most common ADRs (over 10% of total patients) across the groups. Among the ADRs, the frequency of dizziness and nausea were significantly fewer in the dose-titration groups compared with those in the no-titration group. There was no newly reported ADR in our study (Table 3). A total of 11 cases of serious adverse events (SAEs) occurred during the study period (see Table 4). Among the patients, only 2 cases (dizziness and facial bone fracture in group 2; nausea, vomiting, and chilling in group 3) were reported to be possibly/probably related with the study drug (Table 4). There was no death during the study. Drug compliance was similarly good in the three groups (95.9% in group 1, 99.7% in group 2, and 91.5% in group 3; *p* > 0.05).

**Table 3:** Adverse drug reactions during study period (safety population)

Variables	Group 1, 15 mg (*n* = 51)	Group 2, 10/23 mg (*n* = 55)	Group 3, no titration (*n* = 54)	*p* value
Anorexia	9/51, 17.6%	12/55, 21.8%	12/54, 22.2%	0.815
Dizziness*	*4/51, 7.8%*	*4/55, 7.3%*	*12/54, 22.2%*	*0.029*
Nausea*	*4/51, 7.8%*	*5/55, 9.1%*	*13/54, 24.1%*	*0.025*
Vomiting	4/51, 7.8%	7/55, 12.7%	9/54, 16.7%	0.392
Generalized weakness	4/51, 7.8%	8/55, 14.5%	5/54, 9.3%	0.494
Weight loss	4/51, 7.8%	4/55, 7.3%	3/54, 5.6%	0.928
Diarrhea	2/51, 3.9%	4/55, 7.3%	5/54, 9.3%	0.584
Dyspepsia	0/51, 0%	4/55, 7.3%	3/54, 5.6%	0.179
Urinary frequency	2/51, 3.9%	4/55, 7.3%	0/54, 0%	0.122
Increased neuropsychiatric sx.	0/51, 0%	1/55, 1.8%	1/54, 1.9%	1.000
Insomnia	1/51, 2.0%	2/55, 3.6%	0/54, 0%	0.537
Headache	0/51, 0%	0/55, 0%	3/54, 5.6%	0.068
Tremor	1/51, 2.0%	1/55, 1.8%	1/54, 1.9%	1.000
Cold sweating	0/51, 0%	1/55, 1.8%	0/54, 0%	1.000
Drooling	0/51, 0%	1/55, 1.8%	1/54, 1.9%	1.000
Abdominal pain	1/51, 2.0%	0/55, 0%	0/54, 0%	0.319
Unexpected nocturnal ejaculation	0/51, 0%	0/55, 0%	1/54, 1.9%	1.000
Heartburn	0/51, 0%	0/55, 0%	1/54, 1.9%	1.000
PISA syndrome	1/51, 2.0%	0/55, 0%	0/54, 0%	0.319
High BP	0/51, 0%	0/55, 0%	1/54, 1.9%	1.000
Fecal incontinence	0/51, 0%	0/55, 0%	1/54, 1.9%	1.000

*Significantly different incidences (*p* value < 0.05) between titration and no-titration groups

**Table 4:** Summary of serious adverse events (safety population)

Subject	Age	Sex	Study group	Event	Sx. onset (after study initiation)	Relationship to study drug	Seriousness	Dropout
1	78	F	Group 1	Acute cerebral infarct	41 days	Not related	Severe	no
2	78	M	Group 1	Diabetic foot	27 days	Not related	Moderate	no
3	76	M	Group 1	1st seizure (post-stroke)	2 days	Not related	Moderate	yes
4	85	F	Group 2	Fall	47 days	Not related	Mild	yes
5	68	M	Group 2	Unruptured cerebral aneurysm	25 days	Not related	Mild	no
6	76	F	Group 2	Dizziness, facial bone fx.	11 days	Probably related	Moderate	yes
7	78	M	Group 2	Inguinal hernia	2 days	Not related	Moderate	no
8	56	F	Group 3	Lumber sprain	40 days	Not related	Mild	no
9	77	F	Group 3	Influenza A, APN, gastroenteritis	47 days	Not related	Moderate	no
10	81	F	Group 3	Femur fx. after slip down	14 days	Not related	Moderate	no
11	78	F	Group 3	Nausea, vomiting, chilling	0 day	Possibly related	Severe	yes

Relationships between the study drug and AEs were rated as unrelated, possibly related, or probably related

### Relative risks of adverse event

**Fig. 2:**
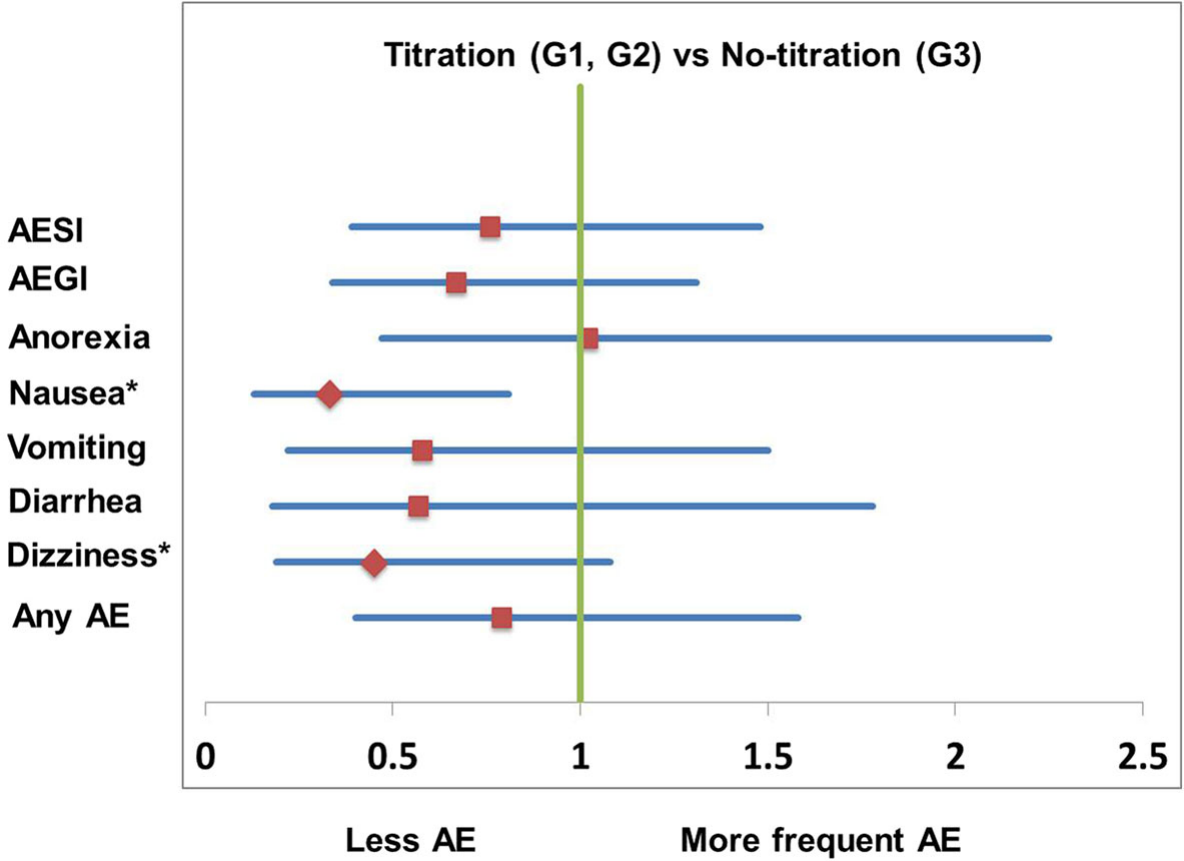
Risk assessments of TEAEs (forest plot using odds ratio)

We measured odds ratio (OR) of each TEAE and compared them between dose-titration groups (groups 1 and 2) and no-titration group (group 3) (Fig. 2). There was no significant difference regarding ORs of each TEAE except nausea and dizziness. Risks of nausea (OR 0.33, confidence level 0.13–0.81) and dizziness (OR 0.45, confidence level 0.19–1.08) in dose-titration groups were significantly lower compared with those in no-titration group (*p* < 0.05; Fig. 2).

## Discussion

In this study, we conducted a 12-week, multicenter, randomized, three-arm prospective clinical trial investigating safety and tolerability of a high-dose donepezil 23 mg according to dose-escalation methods. We also assessed whether the dose titration in the first 4 weeks of the escalation would be preferred to reduce AEs and improve drug adherence in moderate to severe AD patients.

Our study showed three major findings. First, high-dose donepezil was generally tolerable based on that most TEAEs were mild in severities and few SAEs occurred regardless of dose-titration methods. It might be attributed to the property of a slow-release formulation of donepezil 23-mg tablet. Pharmacokinetic analyses investigating donepezil 23 mg compared with donepezil 10 mg have shown pharmacological differences in that the maximum plasma concentration (Cmax) and time to Cmax of the 23-mg dose are approximately twice as much as the 10 mg dose, and its blood levels are maintained over prolonged periods of time. The slower increase of plasma concentration is known to reduce AEs while the higher Cmax with sustained efficacy may increase cognitive benefits [16]. However, dropout rates and incidence of TEAEs were relatively higher than those in other countries [4, 8, 9], which might be explained by the speculation that more cholinergic symptoms and poorer tolerability in non-US populations may be due to lower body weights [9, 10, 17].

Second, cholinergic side effects including anorexia, dizziness, nausea, vomiting, generalized weakness, and diarrhea were the most frequently reported TEAEs of the higher dose, which is consistent with studies using standard dose of 10 mg/day. However, high-dose donepezil showed more cholinergic symptoms compared with standard dose donepezil 10 mg regardless of dose-escalation methods. Our findings are similar to previous reports in that the most common AEs were cholinergic side effects, GI in origin [4, 8–10]. Interestingly, cardiologic symptoms such as bradycardia and syncope as side effects of high-dose donepezil were not reported in any of the groups. This might be explained as several ways; a relatively small sample size considering the few occurrences of bradycardia/ syncope in previous studies [8, 9]; patient’s complaining of other symptoms such as dizziness, generalized weakness, or occurrences of falls; and potential differences between the enrolled patients should be considered.

Third, the incidences of TEAEs were different depending on the dose escalation methods, and a few TEAEs revealed significant differences between dose-titration and no-titration groups. Cholinergic side effects particularly nausea, and possibly dizziness and headache, were fewer in dose-titration groups compared with no-titration group. When considering ADR, the differences became prominent. It may be explained by a decrease of Cmax and an increase of time to maximal plasma concentration (Tmax) which was done by reducing the total daily dose during the first 4 weeks. Despite the slow-releasing formulation, oscillating increase of the plasma drug level after dose escalation and the rapid reach to the Cmax of donepezil 23 mg within the first 4 weeks might have influenced the more frequent AEs in the no-titration group. Hence, it is notable that dose-titration groups showed a better safety in terms of nausea, dizziness, and headache. The results support the idea that dose titration during the first 4 weeks before escalating to 23 mg can avoid a sharp increase in peak concentration and confer benefits in safety and tolerability during the initially critical and the most risky period [7]. Moreover, considering that major AEs leading to premature study discontinuation were nausea, vomiting, dizziness, and generalized weakness in our study, reducing risks of nausea and dizziness would likely enhance drug adherence and finally achieve the expected cognitive benefits by continuing the high-dose donepezil.

Another interesting issue is which titration method would be better regarding patient’s safety and tolerability. Although we could not give a definitive answer to that because there was no statistically significant AE differences between group 1 and group 2, group 1 showed a numerical trend towards fewer TEAEs, fewer dropout rates due to AEs, and fewer moderate to severe AEs, suggesting that 15 mg/day dose-titration might be preferable than alternate dose titration (group 2). Moreover, dropout rates due to AEs in the group 2 were numerically the highest among the groups, which is critically related with drug tolerability, although it did not reach a statistical significance.

To the best of our knowledge, this is the first multi-center trial to investigate safety and tolerability of high-dose donepezil according to the dose-titration methods. In previous studies, in vivo imaging analyses showed insufficient inhibition of cortical AChEI activities of standard dose of donepezil in mild to moderate AD patients; hence, development of high-dose donepezil was supported for optimal responses. Patients with AD, particularly advanced stage, can achieve therapeutic benefits by escalating a dose of donepezil from 10 to 23 mg in case of insufficient or waning cognitive effects despite the stable dose of donepezil 10 mg [4]. Based on the assumption that higher doses of AChEIs might provide greater stabilization and symptomatic improvements in later stages of AD in dose-dependent manner [4], experts recommended escalating to high-dose donepezil when patients with moderate to severe AD dementia showed clinical worsening while receiving a stable dose of donepezil 10 mg, regardless of a memantine combination [16, 18, 19]. At the same time, stepwise dose titration was also recommended before the escalation to enhance a drug adherence [16, 18]. However, there have been no formal clinical trials to back up this argument until this study.

Our study had some limitations. First, the sample size was relatively small. In addition, we only included Korean patients with moderate to severe AD. Considering that safety outcomes in US population were somewhat different from those in non-US population [9], our results need some cautious interpretations to generalize to other populations. Second, we conducted a 12-week safety assessment study, not knowing the long-term safety profile of each dose-titration method. However, based on an open-label extension trial using high-dose donepezil, weight loss was the most commonly reported AE, whereas other TEAEs including bradycardia and GI bleeding were rare during 12 months of donepezil 23 mg and the incidences of TEAEs dropped rapidly after the first 4 weeks [15]. Third, this study was conducted in an open-label design; hence, there might be an expectation bias of titration groups both in investigators and patients. Although the patients were assumed not to remember their group allocation and the side effects according to the titration methods in detail and we tried to overcome the limitation by using the patient’s study number without any information about the group allocation and not revealing the patient’s study group on the medical chart, further trials with blinding might be needed to confirm our results. Additionally, larger dropout rates than previous trials might be affected by the open-label design. However, we assessed causes of premature study discontinuations and included the dropout population for safety analyses to prevent misinterpretations. Lastly, we could not provide drug efficacy data comparing dose-titration with no-titration groups because we did not measure efficacy outcomes in favor of safety and tolerability as per the study plan.

Despite these several limitations in generalizing our results, this is the first evidence to support the experts’ opinion and the value of dose titration before escalating to high-dose donepezil 23 mg. Further trials with a larger sample size, long-term follow-ups, and extensive outcome measures would be needed to confirm the benefits of dose titration in moderate to severe AD.

## Conclusion

In this study, titration for escalating to 23 mg of high-dose donepezil showed better safety in terms of cholinergic side effects including nausea, dizziness, and headache. These findings suggest that dose titration during the first 4 weeks can be recommended to enhance safety for patients with moderate to severe AD who require high-dose donepezil.

## Additional file

Additional file 1: **Table S1.** Baseline demographics and clinical characteristics of the subjects (per protocol population). Table S2. Comparisons of baseline demographics and clinical characteristics between study completers and dropout patients. (DOCX 20 kb)

## Abbreviations

AChEI: Acetylcholinesterase inhibitor
AD: Alzheimer’s disease
ADR: Adverse drug reactions
AE: Adverse events
AESI: Adverse events of special interests
ANOVA: Analysis of variance
CDR: Clinical dementia rating
Cmax: Maximum plasma concentration
MMSE: Mini-Mental State Examination
OR: Odds ratio
PP: Per-protocol
SAE: Serious adverse events
TEAE: Treatment emergent adverse events
Tmax: Time to maximal plasma concentration
GI: Gastrointestinal
