# Supplementary material for: Safety and tolerability of donepezil 23 mg with or without intermediate dose titration in patients with Alzheimer’s disease taking donepezil 10 mg: a multicenter, randomized, open-label, parallel-design, three-arm, prospective trial
Source: Alzheimers Res Ther. 2019 May 1;11:37. doi: 10.1186/s13195-019-0492-1 (PMC6492390; doi:10.1186/s13195-019-0492-1)
Supplement: Supplementary file 1 — Table S1. Baseline demographics and clinical characteristics of the subjects (per protocol population). Table S2. Comparisons of baseline demographics and clinical characteristics between study completers and dropout patients. (DOCX 20 kb) [file 13195_2019_492_MOESM1_ESM.docx]

**Table S1.** Baseline demographics and clinical characteristics of the subjects (per protocol population)

| Variables | Group 1,15mg (n=38) | Group 2,10/23mg (n=36) | Group 3,no titration (n=36) | *P* |
| --- | --- | --- | --- | --- |
| Age, yrs | 74.9±8.7 | 73.1±10.8 | 76.2±7.9 | 0.663* |
| Female, % | 60.5% | 52.8% | 58.3% | 0.787 |
| Education, low/intermediate/high | 50%/28.9%/21.1% | 47.2%/25%/27.8% | 50%/25%/25% | 0.970 |
| K-MMSE | 13.2±4.9 | 14.8±4.1 | 14.3±4.8 | 0.399* |
| CDR | 1.6±0.6 | 1.5±0.6 | 1.9±0.3 | 0.056* |
| GDS | 4.8±0.6 | 4.7±0.7 | 4.9±0.7 | 0.533* |
| Body weight, kg | 58.5±8.8 | 60.2±12.1 | 58.6±9.7 | 0.736 |
| BMI | 24.2±2.6 | 24.0±3.7 | 23.9±3.2 | 0.947 |
| Duration of donepezil, yrs | 1.4±1.2 | 2.0±1.7 | 1.7±2.1 | 0.146* |
| History of side effect of donepezil, % | 2/38, 5.3% | 2/36, 5.6% | 0/36, 0% | 0.544 |
| Hypertension, % | 17/38, 44.7% | 19/36, 52.8% | 15/36, 41.7% | 0.620 |
| DM, % | 9/38, 23.7% | 10/36, 27.8% | 8/36, 22.2% | 0.851 |
| Hyperlipidemia, % | 14/38, 36.8% | 12/36, 33.3% | 13/36, 36.1% | 0.947 |
| Brain injury, % | 3/38, 7.9% | 4/36, 11.1% | 4/36, 11.1% | 0.854 |

*Kruskal-Wallis method. Educational level up to 6 years (elementary school) was rated as low, up to 12 years (graduation high school) was rated as medium, and above 12 years was rated as high.

**Table S2.** Comparisons of baseline demographics and clinical characteristics between study completers and drop out patients

| Variables | Completers (n=110) | Drop out patients (n=50) | *P* |
| --- | --- | --- | --- |
| Age, yrs | 78.0±7.1 | 77.7±6.8 | 0.427* |
| Female, % | 63/110, 57.3% | 35/50, 70.0% | 0.126 |
| Education, low/intermediate/high | 54/29/27 | 30/12/8 | 0.368 |
| K-MMSE | 14.1±4.6 | 12.8±5.0 | 0.116 |
| CDR | 1.7±0.6 | 1.6±0.7 | 0.459* |
| GDS | 5.1±0.5 | 5.2±0.6 | 0.213* |
| Body weight, kg | 59.1±10.2 | 56.2±9.6 | 0.093 |
| BMI | 24.0±3.2 | 23.5±3.2 | 0.351 |
| Duration of donepezil, yrs | 1.5±1.7 | 2.4±3.0 | 0.733* |
| Hypertension, % | 51/110, 46.4% | 25/50, 50% | 0.669 |
| DM, % | 27/110, 24.5% | 12/50, 24% | 0.941 |
| Hyperlipidemia, % | 39/110, 35.5% | 18/50, 36% | 0.947 |
| Brain injury, % | 11/110, 10% | 2/50, 4% | 0.348 |

*Mann-Whitney U test. Educational level up to 6 years (elementary school) was rated as low, up to 12 years (graduation high school) was rated as medium, and above 12 years was rated as high.
